# *Fusobacterium nucleatum* upregulates MMP7 to promote metastasis-related characteristics of colorectal cancer cell via activating MAPK(JNK)-AP1 axis

**DOI:** 10.1186/s12967-023-04527-3

**Published:** 2023-10-09

**Authors:** Suwen Ou, Haipeng Chen, Hufei Wang, Jinhua Ye, Huidi Liu, Yangbao Tao, Songlin Ran, Xiaoqin Mu, Fangzhou Liu, Shuang Zhu, Kangjia Luo, Zilong Guan, Yinghu Jin, Rui Huang, Yanni Song, Shu-lin Liu

**Affiliations:** 1 Department of Colorectal Surgery, The Second Affiliated Hospital of Harbin Medical University, Harbin, 150081 China; 2 Department of Colorectal Surgery, National Clinical Research Center of Cancer/Cancer Hospital, National Cancer Center, Chinese Academy of Medical Sciences and Peking Union Medical College, Beijing, 100021 China; 3 Genomics Research Center (Key Laboratory of Gut Microbiota and Pharmacogenomics of Heilongjiang Province), College of Pharmacy, Harbin Medical University, Harbin, 150081 China; 4 Cumming School of Medicine Centre for Infection and Genomics, Harbin Medical University-University of Calgary, Harbin Medical University, Harbin, 150081 China; 5 Department of Gastrointestinal Surgery, The Affiliated Hospital of Medical School of Ningbo University, Ningbo, 315020 China; 6 Department of General Surgery, The Fourth Affiliated Hospital of Harbin Medical University, Harbin, 150000 China; 7 Department of Breast Surgery, Harbin Medical University Cancer Hospital, Harbin, 150081 China; 8 Department of Microbiology, Immunology and Infectious Diseases, University of Calgary, Calgary, AB T2N 4N1 Canada; 9 Department of Pancreatic and Biliary Surgery, The First Affiliated Hospital of Harbin Medical University, Harbin, 150001 China

**Keywords:** Colorectal cancer, *Fusobacterium nucleatum*, Matrix metalloproteinase 7, Migration

## Abstract

**Background:**

Colorectal cancer (CRC) is the third most common malignant tumor. *Fusobacterium nucleatum* (*F. nucleatum*) is overabundant in CRC and associated with metastasis, but the role of *F. nucleatum* in CRC cell migration and metastasis has not been fully elucidated.

**Methods:**

Differential gene analysis, protein−protein interaction, robust rank aggregation analysis, functional enrichment analysis, and gene set variation analysis were used to figure out the potential vital genes and biological functions affected by *F. nucleatum* infection. The 16S rDNA sequencing and q-PCR were used to detect the abundance of *F. nucleatum* in tissues and stools. Then, we assessed the effect of *F. nucleatum* on CRC cell migration by wound healing and transwell assays, and confirmed the role of Matrix metalloproteinase 7 (MMP7) induced by *F. nucleatum* in cell migration. Furthermore, we dissected the mechanisms involved in *F. nucleatum* induced MMP7 expression. We also investigated the MMP7 expression in clinical samples and its correlation with prognosis in CRC patients. Finally, we screened out potential small molecular drugs that targeted MMP7 using the HERB database and molecular docking.

**Results:**

*F. nucleatum* infection altered the gene expression profile and affected immune response, inflammation, biosynthesis, metabolism, adhesion and motility related biological functions in CRC. *F. nucleatum* was enriched in CRC and promoted the migration of CRC cell by upregulating MMP7 in vitro. MMP7 expression induced by *F. nucleatum* infection was mediated by the MAPK(JNK)-AP1 axis. MMP7 was highly expressed in CRC and correlated with CMS4 and poor clinical prognosis. Small molecular drugs such as δ-tocotrienol, 3,4-benzopyrene, tea polyphenols, and gallic catechin served as potential targeted therapeutic drugs for *F. nucleatum* induced MMP7 in CRC.

**Conclusions:**

Our study showed that *F. nucleatum* promoted metastasis-related characteristics of CRC cell by upregulating MMP7 via MAPK(JNK)-AP1 axis. *F. nucleatum* and MMP7 may serve as potential therapeutic targets for repressing CRC advance and metastasis.

**Supplementary Information:**

The online version contains supplementary material available at 10.1186/s12967-023-04527-3.

## Introduction

Colorectal cancer (CRC) ranked as the third in new cases of malignant tumors and was the second leading cause of cancer death in 2020 [1]. About 25% of CRC patients present with metastatic lesions at the time of diagnosis, and more than 50% of CRC patients will develop metastases eventually [2]. The 5-year survival rate of patients with distant metastasis is less than 10% [3]. Therefore, it is of great significance to uncover the mechanism and causes of CRC metastasis for the prevention and treatment of CRC.

Microbes are involved in the development of a large number of cancers. It is conservatively estimated that about 15% of cancer cases in the world can be attributed to the infection of pathogens [4]. The composition of mucosal microbiota has changed during the development of colorectal cancer, indicating that some gut bacteria may play a role in the occurrence and development of CRC [5]. In the past decade, a rising number of researches focused on the role of bacteria in the evolution of colorectal cancer [6]. *Fusobacterium nucleatum* (*F. nucleatum*) is gram-negative anaerobic bacillus, which is enriched not only in feces but also in the tumor tissues of CRC patients [7–9]. It has been reported that *F. nucleatum* not only promotes the proliferation of CRC cells [10] but also enhances the resistance of CRC cells to chemotherapy [11, 12]. In addition, the abundance of *F. nucleatum* in CRC tissue is also related to metastasis and prognosis [13]. Recent studies have shown that *F. nucleatum* can promote CRC cell metastasis by regulating epithelial mesenchymal transformation, non-coding RNA expression, exosomes production and secretion, m6A modification of mRNA, etc. [14–17]. However, the exact roles and potential mechanisms of *F. nucleatum* promoting CRC metastasis remain largely unclear.

Matrix metalloproteinases (MMPs) family plays an important role in tumor matrix degradation and remodeling. MMPs can degrade various protein components in extracellular matrix (ECM), destroy the histological barrier of cancer invasion, and play a key role in cancer cell invasion and metastasis [18]. In this study, we found that *F. nucleatum* infection promoted CRC cell migration by upregulating MMP7 via activating the MAPK (JNK)-AP1 axis. Our data provide new insights into the role of *F. nucleatum* in colorectal cancer metastasis and assist translational research in using *F. nucleatum* and MMP7 as potential therapeutic targets for the treatment of CRC.

## Materials and methods

### Public data source

The transcriptome data of CRC cell lines (LoVo and Caco-2) were downloaded from Gene Expression Omnibus, with accession ID: GSE173549 and GSE102573. The gene expression data and clinical information of TCGA COAD and READ cohorts were obtained from UCSC database [19]. Then, the RNA sequencing data were converted to transcripts per kilobase million (TPM) and further transformed to log_2_(TPM + 1). The combined GEO cohort was integrated by five microarray data sets of GPL570 platform (GSE39582, GSE14333, GSE17536, GSE17537, and GSE72968). Background correction, standardization, and batch effect were described in our previous study [20]. Patients with survival time less than 1 month were removed during survival analysis. Kaplan–Meier (KM) plots were visualized using the “survminer” package. Statistical significance was evaluated by Log-rank test. And in this study, R package “CMScaller” was used for the Consensus Molecular Subtypes (CMS) classification as described by the original author [21].

### Differential expression analysis

R package “limma” was used for performing the differential expression analysis [22]. The differentially expressed genes (DEGs) were identified through adjusted *P* < 0.05 and |log2FC| ≥ 1. The volcano and heat maps were visualized using the “ggplot2” and “pheatmap” packages, respectively.

### Protein−protein interaction analysis and visualization

Upregulated genes by *F. nucleatum* infection in both CRC cell lines LoVo and Caco-2 were inputted into online bioinformatics tool STRING (https://cn.string-db.org/) to conduct Protein−Protein Interaction Analysis. The result of Interaction Network was visualized by Cytoscape Software (v3.9.1).

### Robust rank aggregation analysis

The Robust Rank Aggregation (RRA) can rank and identify the most robust DEGs among multiple datasets [23]. In GSE173549 and GSE102573, genes with adjusted P < 0.05 and |log2FC| ≥ 0.5 were integrated using the R package “RobustRankAggreg” to find robust DEGs. Then, the top 10 upregulated and downregulated DEGs ranked by RRA were visualized via a heat map.

### Functional enrichment analysis

Gene Ontology (GO) and Kyoto Encyclopedia of Genes and Genomes (KEGG) analyses were performed with DEGs meeting adjusted P < 0.05 and |log2FC| ≥ 1 using the R package “clusterProfiler” [24, 25]. The top 15 enrichment terms of GO and KEGG were visualized using the above package. Furthermore, we downloaded hallmark gene sets that summarize and represent specific well-defined biological states or processes from MSigDB website [26]. Then, Gene Set Variation Analysis (GSVA) was used to investigate the differences in hallmark gene sets between *F. nucleatum*-infected and non-infected cells [27]. Gene Set Enrichment Analysis (GSEA) was applied to explore the biological functions influenced by *F. nucleatum* in our own transcriptome data using the C5 gene sets of MSigDB website. The significant terms of GSEA were visualized using the R package “enrichplot”.

### Correlation analysis

The Pearson correlation analysis was performed with “ggpubr” package and visualized using “ggplot2” package. The representative genes of KEGG MAPK signaling pathway were downloaded from MSigDB website, and ssGSEA algorithm was then used to calculate the KEGG MAPK signaling pathway scores of CRC patients.

### Identification of components targeting MMP7 and network visualization

We used the HERB database [28] to find components that target MMP7. Components-MMP7 network map was constructed using Cytoscape Software.

### Molecular docking

Using molecular docking analysis, we predicted the binding of proteins, along with the free energy of binding, and differential components. The molecular structures of these components were obtained from the PubChem database [29] and the protein structures of MMP7 (PDB ID:7WXX) were obtained from the PDB database (https://www.rcsb.org/) [30]. Molecular docking was performed using AutoDock Tools software (1.5.7). A heat map was plotted by Sangerbox online drawing tool, and PYMOL software was used for the visualization of optimal docking results.

### Bacterial strain and cell culture

*F. nucleatum* (ATCC 25,586) was purchased from Guangdong Microbial Culture Collection Center (GDMCC), and grown in Brain Heart Infusion Broth (hopebio, China) supplemented with hemin, K_2_HPO_4_, Vitamin K1, and L-Cysteine in a round bottom vertical anaerobic culture bag (hopebio, China) at 37 ℃. *Escherichia coli* strain DH5α (obtained from Genomics Research Center, College of Pharmacy, Harbin Medical University) was cultured in Luria-Bertani medium. Human CRC cell lines (HCT-116, LoVo) and a normal colon epithelium cell line (NCM460) were purchased from the American Type Culture Collection (ATCC, USA). HCT116 and LoVo were cultured in RPMI 1640 (Gibco, USA) and F-12K Nutrient Mixture (Gibco, USA) supplemented with 10% fetal bovine serum (purchased from Inner Mongolia Opcel Biotechnology Co.,Ltd., China), respectively. All cell lines were cultured at 37 ℃ in humidified 5% CO_2_ atmosphere.

### Clinical samples

Stool samples were collected from 26 healthy volunteers, 13 patients with acute abdominal diseases, 18 patients with colorectal precancerous lesion (adenomas or polyps) and 76 patients with CRC. All patients did not undergo surgical treatment before collecting fecal specimens. Colorectal cancer and adjacent normal tissues were obtained from surgical specimens of 19 patients diagnosed with CRC. All specimens were collected during 2019–2022. All participants were from the Second Affiliated Hospital of Harbin Medical University (Harbin, China).

### DNA extraction and 16S rRNA gene sequencing

Genomic DNA (gDNA) was extracted from fresh frozen colorectal cancer tissue with the E.Z.N.A.^®^ tissue DNA Kit (Omega Bio-tek, U.S.) and from fecal samples with the E.Z.N.A.^®^ soil DNA Kit (Omega Bio-tek, U.S.) according to manufacturer’s instructions. The V3-V4 regions of the 16S rRNA gene were PCR amplified using primer pairs 338F (5′-ACTCCTACGGGAGGCAGCAG-3′) and 806R (5′ GGACTACHVGGGTWTCTAAT-3′). The PCR reaction was set up in triplicate, and the PCR product was purified using the AxyPrep DNA Gel Extraction Kit (Axygen Biosciences, USA). The high-throughput sequencing was performed in Majorbio Bio-Pharm Technology Co. Ltd. (Shanghai, China). Analysis of the 16S rRNA microbiome sequencing data was performed using the free online platform of Majorbio Cloud Platform (cloud.majorbio.com).

### ***F. nucleatum *** quantification

Genomic DNA extraction from fresh frozen colorectal tissue was conducted as described above. gDNA from each specimen was subjected to qPCR to estimate the abundance of *F. nucleatum* by detecting the 16S rRNA gene. Each reaction was assayed in triplicate in 20 µl reaction containing PowerUp SYBR Master Mix (Applied Biosystems, USA), primers, template DNA, ddH_2_O, and was placed in an optical PCR plate. Amplification and detection of DNA were performed with the ABI StepOne^™^ Real-Time PCR System (Applied Biosystems, USA) under the following reaction conditions: 10 min at 95 ℃, followed by 40 cycles of denaturation at 95 ℃ for 15 s and at 60℃ for 1 min. The cycle threshold (Ct) values for *F. nucleatum* were normalized to the amount of human biopsy gDNA in each reaction by using PGT as a reference gene. The following primer sets were used:

*F. nucleatum*:

Forward: 5ʹ-CGGGTGAGTAACG CGTAAAG-3ʹ,

Reverse: 5ʹ-GCATTCGTTTCCAAATGTTGTCC-3ʹ;

PGT

Forward: 5ʹ-ATCCCCAAAGCACCTGGTTT-3ʹ,

Reverse: 5ʹ-AGAGGCCAAGATAGTCCTGGTAA-3ʹ.

### RNA sequencing

LoVo cells were cocultured with PBS or *F. nucleatum* at a multiplicity of infection (MOI) of 100:1 for 2 h. Total RNA was extracted from the treated cells using TRIzol^™^ Reagent according the manufacturer’s instructions (Invitrogen, USA) and genomic DNA was removed using DNase I (TaKara, Japan). Then RNA quality was determined by 2100 Bioanalyser (Agilent, USA) and quantified using the ND-2000 (NanoDrop Technologies, USA). Only high-quality RNA sample (OD260/280 = 1.8 ~ 2.2, OD260/230 ≥ 2.0, RIN ≥ 6.5, 28S:18S ≥ 1.0, > 1 µg) was used to construct sequencing library. RNA-seq transcriptome library was prepared following TruSeqTM RNA sample preparation Kit (Illumina, USA) using 1 µg of total RNA. Messenger RNA was isolated according to polyA selection method by oligo (dT) beads and then fragmented by fragmentation buffer. Double-stranded cDNA was synthesized using a SuperScript double-stranded cDNA synthesis kit (Invitrogen, USA) with random hexamer primers (Illumina, USA). Then the synthesized cDNA was subjected to end-repair, phosphorylation and ‘A’ base addition according to Illumina’s library construction protocol. Libraries were size selected for cDNA target fragments of 300 bp on 2% Low Range Ultra Agarose followed by PCR amplified using Phusion DNA polymerase (NEB, USA) for 15 PCR cycles. After quantification by TBS380, paired-end RNA-seq sequencing library was sequenced with the NovaSeq 6000 sequencer (2 × 150 bp read length). Paired-end clean reads were aligned to the human genome version hg38 using Hisat2 v2.0.5. Differential expression analysis of two groups was performed using the DESeq2. Differences with P value < 0.05 and | log2 (fold change) | > 1 were considered as statistically significant.

### RNA extraction and quantitative real-time PCR

Total RNAs were extracted from CRC cell lines by using TRIzol™ Reagent (Invitrogen, USA). 1 µg of total RNAs was reverse transcribed to cDNA using PrimeScript^™^ RT Master Mix (Takara, Japan). Quantitative real-time PCR was performed in ABI StepOne^™^ Real-Time PCR System. Each reaction was assayed in quadruplicate in 20 µl reaction containing PowerUp SYBR Master Mix (Applied biosystems, USA), primers, template cDNA and ddH_2_O. Relative abundance of mRNA was calculated by 2 − ΔΔCt method. ACTB served as internal reference gene. The following primer sets were used:

Human MMP7:

Forward: 5ʹ- CATGATTGGCTTTGCGCGAG-3ʹ,

Reverse: 5ʹ- AGACTGCTACCATCCGTCCA-3ʹ;

Human ACTB:

Forward: 5ʹ- GATTCCTATGTGGGCGACGA-3ʹ,

Reverse: 5ʹ- AGGTCTCAAACATGATCTGGGT-3ʹ;

### Wound healing assays

CRC cells were seeded in 6-well plates to create a confluent monolayer. The cells of each well were incubated with *F. nucleatum* or *E. coli* (DH5α) at a MOI of 100:1, or equal volume of PBS for 2 h. The cell monolayers were then scraped with 10 µl pipette tips in a straight line to create a scratch. After washing twice with PBS, the cells were incubated in serum-free medium supplemented with penicillin, streptomycin, metronidazole for 24 h. Cell scratch images were taken under a microscope at 0, 24 h, respectively. The scratch area was measured by ImageJ software. The cell migration rate (%) = (0 h scratch area − 24 h scratch area)/ 0 h scratch area ×100%.

### Transwell migration assays

CRC cell lines were incubated with PBS, *F. nucleatum* or *E. coli* for 2 h in advance. For migration assay, 1 × 10^5^ cells suspended in 200 µl medium supplemented with 1% fetal bovine serum were seeded in the upper chamber of transwell chambers (8 μm pores, Corning, USA), and 800 µl fresh medium with 10% serum was added to the lower chamber. After incubation for 36 h at 37 °C, the cells in the upper chamber were fixed with 4% paraformaldehyde, followed by staining with 0.1% crystal violet. The images in five fields (100×) were taken under an optical microscope. The migrated cells were quantified by using ImageJ software.

### Protein extraction and western blotting

Total proteins were extracted from CRC cell lines by using RIPA lysis buffer (Beyotime, China) supplemented with PMSF (Beyotime, China) and phosphatase inhibitor (Roche, Switzerland) and then quantified using BCA Protein Assay Kit (Beyotime, China). Nuclear and cytoplasmic protein were prepared using the Nuclear and Cytoplasmic Protein Extraction Kit (Absin, China) according to the protocol provided by the manufacturer. Protein was electrophoresed through 10% SDS polyacrylamide gels and then transferred to PVDF membranes. The membranes were blocked with 5% fat-free milk for 2 h and incubated with primary antibodies at 4 °C overnight. Membranes were then incubated with second antibodies labeled with HRP at room temperature for 2 h on the following day and the signal was detected using an ECL kit (Beyotime, China). The following primary antibodies were used: JNK (Abcam # ab179461), P-JNK (Abcam # ab124956), c-Jun (CST# 9165), P-c-Jun (Abcam#ab32385), and MMP7 (Abcam#ab205525). β-actin (Abclonal#AC026), GAPDH (Abclonal#AC002) or PCNA (Abcam#ab29) was used as reference gene.

### Transcription factors prediction

The four online bioinformatics tools AnimalTFDB3 (http://bioinfo.life.hust.edu.cn/AnimalTFDB/), TRANSFAC PATCH (http://gene-regulation.com/cgi-bin/pub/programs/patch/bin/patch.cgi), TRANSFAC MATCH (http://gene-regulation.com/cgi-bin/pub/programs/match/bin/match.cgi), and PROMO (https://alggen.lsi.upc.es/) were used for scanning the potential transcription factors of MMP7. Binding score of five selected transcription factors was evaluated by using JASPAR (http://jaspar.genereg.net/).

### Lentivirus production and cell transfection

MMP7 shRNA lentivirus and their control shRNA lentivirus were generated using the GV344 (hU6-MCS-Ubiquitin-firefly_Luciferase-IRES-puromycin) vector (Genechem Co. Ltd., Shanghai, China). JUN shRNA lentivirus and control shRNA lentivirus were generated using the GV248 (hU6-MCS-Ubiquitin-EGFP-IRES-puromycin) vector (Genechem Co. Ltd., Shanghai, China). Transfection of the lentivirus construct was performed according to the manufacturer’s instructions. Following transfection, cells stably expressing shRNAs were selected in the presence of puromycin (Beyotime, China) for 5 days. The efficiencies of shRNA knockdown were confirmed with qPCR and western blotting.

### Immunofluorescence

CRC cells were seeded on chamber slides and cocultured with PBS or *F. nucleatum* (MOI = 100:1) for 2 h. After washing with PBS, the cells were fixed with 4% paraformaldehyde for 15 min at room temperature. The slides were incubated with 0.2% Triton X-100 for 10 min and blocked in 3% BSA for 30 min. After discarding the blocking solution, the slides were incubated with primary antibody against c-Jun (CST# 9165T) at room temperature for 2 h. Then the slides were washed by PBST three times and incubated with secondary antibody conjugated with Alexa Fluor^®^ 488 (CST#4412S) and DAPI (Beyotime, China) at room temperature for 1 h and 5 min, respectively. The slides were fixed with Antifade Mounting Medium (Beyotime, China) and imaged using light microscopy.

### Statistical analysis

All bioinformatic analyses were performed in R 4.0.3. Kruskal-Wallis test, Mann Whitney U test, Wilcoxon test, Welch t’ test, Paired t test, Student’s t test, one-way ANOVA, and the log-rank test were used to analyze the data. All statistical tests were performed using GraphPad Prism Software. Data were shown as mean ± standard deviation (SD). P < 0.05 was considered statistically significant.

## Result

### ***F. nucleatum*** infection affects gene expression profile and biological function of colorectal cancer cell

**Fig. 1:**
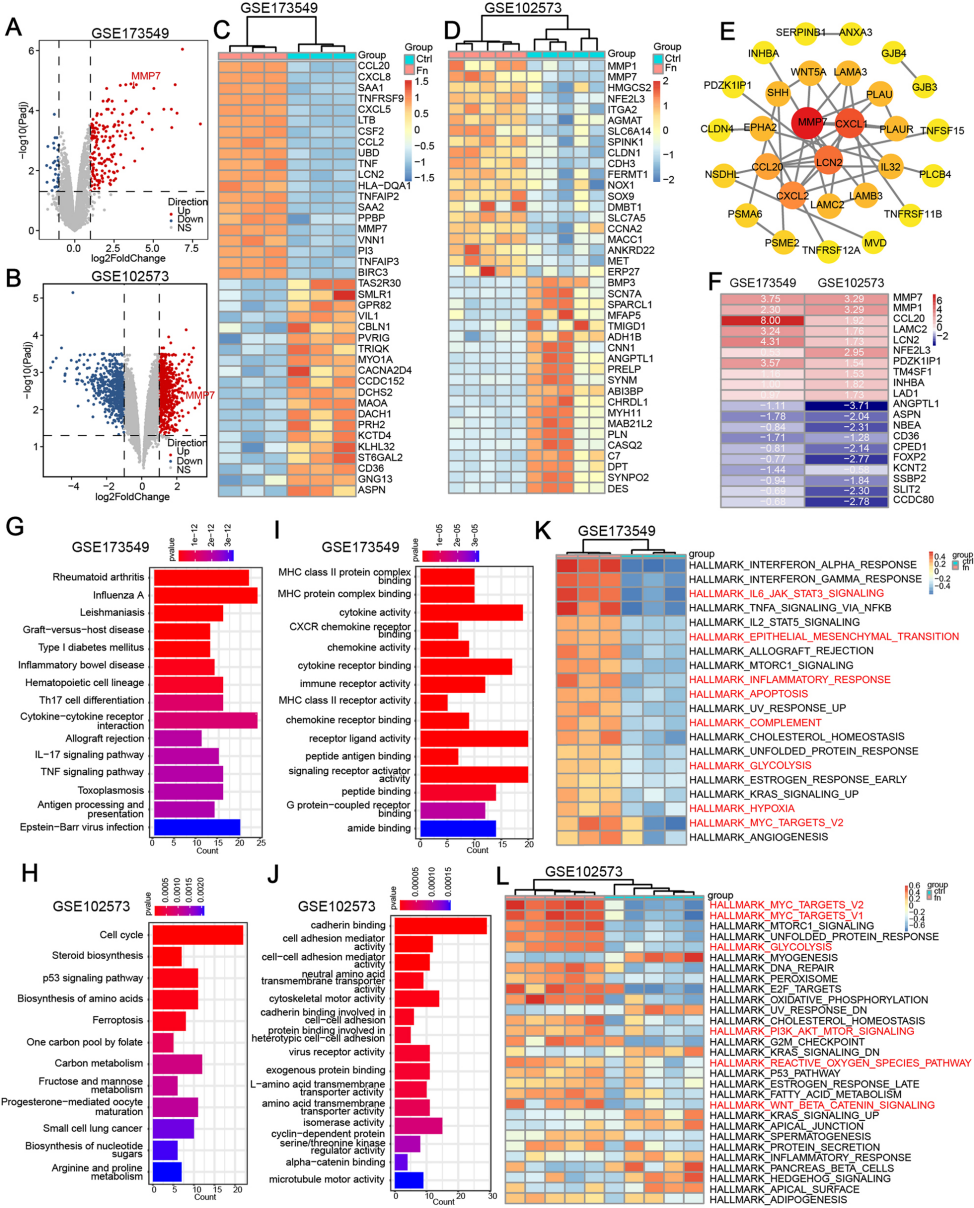
* F. nucleatum* infection affects gene expression profile and biological function of colorectal cancer cell. **A–D** Volcano plots (**A**, **B**) and Heat maps (**C**, **D**) representing the differentially expressed genes between *F. nucleatum*-infected and PBS-treated colorectal cancer cells (GSE173549, LoVo; GSE102573, Caco-2). **E** Protein−protein interaction network of genes upregulated in both two cell lines. Network nodes represent proteins, and Edges represent protein−protein associations. **F** The top upregulated and downregulated genes by *F. nucleatum* infection via RRA analysis. **G**, **H** The activated pathways by *F. nucleatum* infection via KEGG enrichment analysis. **I**, **J** The biological functions influenced by *F. nucleatum* infection via GO enrichment analysis. **K**, **L** The hallmarks of CRC cell affected by *F. nucleatum* infection via GSVA

To look into the overall impact of *F. nucleatum* infection on the biological properties of colorectal cancer, we analyzed the public transcriptome data of CRC cell lines (LoVo and Caco-2) with or without *F. nucleatum* infection. Through differential expression analysis, we obtained 206 differentially expressed genes in LoVo GSE173549 (175 upregulated, 31 downregulated) and 1208 in Caco-2 GSE102573 (605 upregulated, 603 downregulated) affected by *F. nucleatum* infection (Fig. 1A, B). The heat maps show the top 20 upregulated genes and 20 downregulated genes (Fig. 1C, D). Among them, CCL20 and CXCL8, both of which could promote the migration and metastasis of colorectal cancer cells, were validated to be upregulated by *F. nucleatum* infection [15, 31]. BIRC3 was reported to be upregulated by *F. nucleatum* infection and mediate the *F. nucleatum*-induced chemoresistance in CRC cells (Fig. 1C) [12]. As a vital gene related to CRC invasion and metastasis [18], MMP7 is upregulated significantly by *F. nucleatum* in both two datasets. We conduct protein−protein interaction analysis with the common genes significantly upregulated by *F. nucleatum* infection in both datasets. The result indicates that MMP7, CXCL1, and LCN2 are the core node in the whole protein−protein interaction network (Fig. 1E). The heat map shows the top 10 genes of RRA analysis in the two datasets (Fig. 1F). MMP7, MMP1, and CCL20 are the most obviously changed genes in the CRC cells infected by *F. nucleatum*. KEGG pathway enrichment and GO analyses were used to investigate the *F. nucleatum* affected biological function of CRC cell. The results of KEGG analysis revealed that *F. nucleatum* mainly altered the immune response, inflammation and infection related signal pathways in GSE173549 (Fig. 1G), and altered the biosynthesis, metabolism, cell cycle, and ferroptosis pathways in GSE102573 (Fig. 1H). According to GO analysis, *F. nucleatum* infection primarily affected the functions of cell surface receptor-ligand binding involved in MHC protein complex, chemokine, cytokine, and their receptors in GSE173549 (Fig. 1I), and affected the cell functions of adhesion and motility, which were important for metastasis in GSE102573 (Fig. 1J). GSVA was used to further analysis the impact of *F. nucleatum* on CRC. Some common hallmarks were up-regulated by *F. nucleatum* infection in both two datasets, such as MYC _TARGETS, GLYCOLISIS, MTORC1_SIGNALING, and KRAS_SIGNALING (Fig. 1K, L). However, some hallmarks were up-regulated only in GSE173549, such as EPITHELIAL_MESENCHYMAL_TRANSITION, INFLAMMATORY_RESPONSE, COMPLEMENT, and HYPOXIA (Fig. 1K), or only in GSE173549, such as PI3K_AKT_MTOR_SIGNALING, REACTIVE_OXYGEN_SPECIES_PATHWAY, and WNT_BETA_CATENIN_SIGNALING (Fig. 1L).

### ***F. nucleatum *** is overabundant in CRC and promotes the migration of CRC cells

**Fig. 2:**
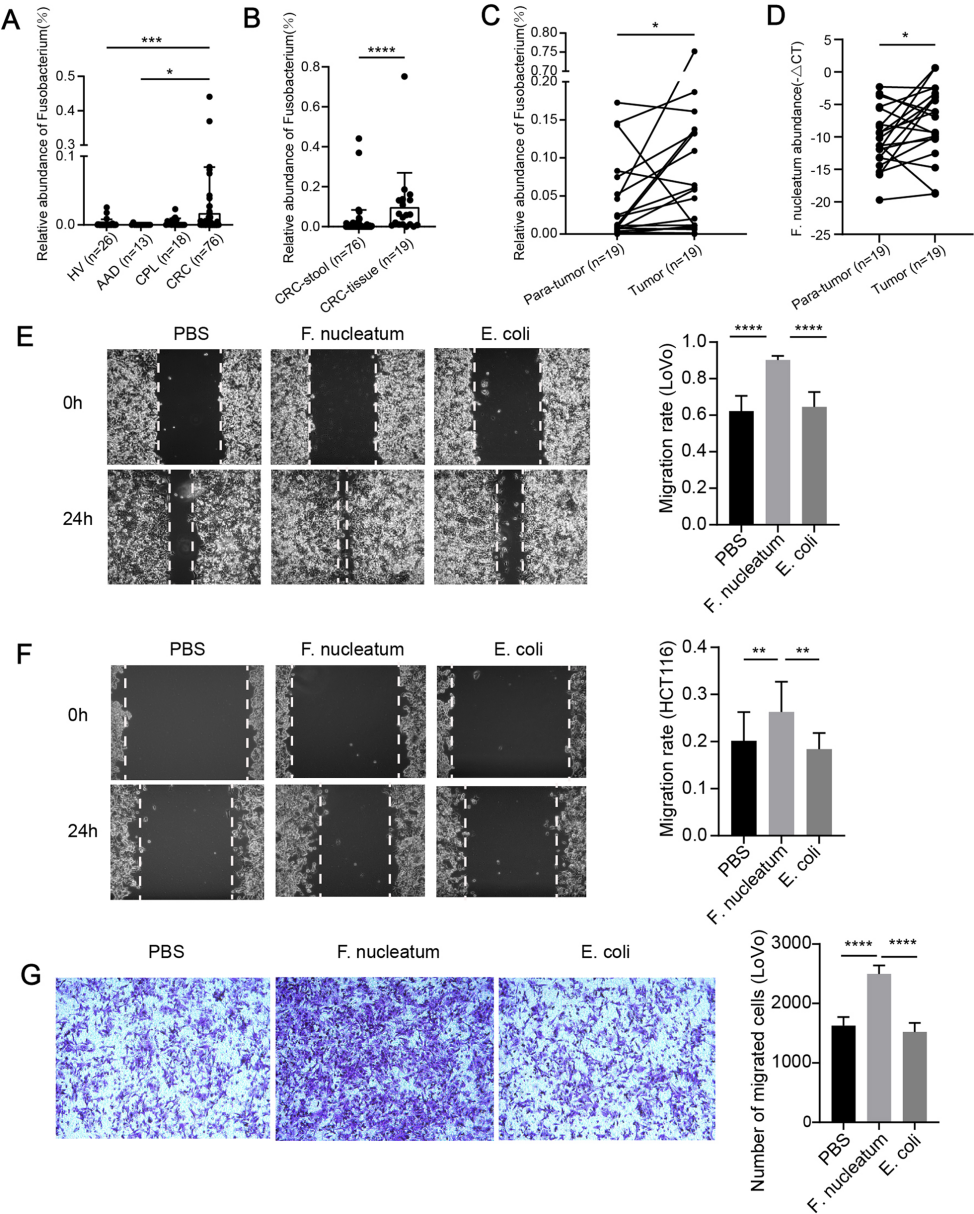
* F. nucleatum* is overabundant in CRC and promotes the migration of CRC cells. **A** The relative abundance of fecal *Fusobacterium* spp. among the healthy volunteers (HV, n = 26), the patients with acute abdominal diseases (AAD, n = 13), the patients with colorectal precancerous lesion (CPL, n = 18), and the patients with colorectal cancer (CRC, n = 76, Kruskal-Wallis test). **B** The relative abundance of *Fusobacterium* spp. in stool (n = 76) and cancer tissue of colorectal cancer (n = 19, Mann Whitney U test). **C** The relative abundance of *Fusobacterium* spp. in paired cancer tissue and adjacent normal tissue (Wilcoxon test). **D** The abundance of *F. nucleatum* in paired cancer tissue (n = 19) and adjacent normal tissue (n = 19, Wilcoxon test). **E**, **F** the motility of LoVo and HCT116 cells was detected by wound-healing assay after incubation with PBS or *F. nucleatum* or *E. coli* (n = 6, one-way ANOVA). **G** the motility of LoVo was tested by transwell assay after treatment with PBS, *F. nucleatum* or *E. coli* (n = 5, one-way ANOVA). Data are shown as mean ± SD, * p < 0.05, ** p < 0.01, *** p < 0.001, **** p < 0.0001

To assess the association between *F. nucleatum* and CRC, we detected *Fusobacterium* spp. abundance in feces and tissues of healthy volunteers and patients using 16S ribosomal RNA gene sequencing. We found that the abundance of *Fusobacterium* spp. in feces from colorectal cancer patients was significantly higher than healthy volunteers and patients with acute abdominal diseases (Fig. 2A). The abundance of *Fusobacterium* spp. in CRC tissues was significantly higher than feces from CRC patients (Fig. 2B). In paired tissue samples, the abundance of *Fusobacterium* spp. in cancer tissues was significantly higher than adjacent normal tissues (Fig. 2C). Consistently, the result of qPCR showed that the abundance of *F. nucleatum* in cancer tissues was significantly higher than adjacent normal tissues (Fig. 2D). This result was also consistant with the privious research [10]. In addition, we evaluated the effect of *F. nucleatum* on the migration of CRC cells with wound-healing and transwell assays. CRC cell lines HCT116 and LoVo infected with *F. nucleatum* had enhanced migratory capacity compared to cells uninfected (PBS) or infected with common intestinal bacteria *E. coli* (DH5α) (Fig. 2E–G).

### ***F. nucleatum *** upregulates MMP7 in CRC cells

**Fig. 3:**
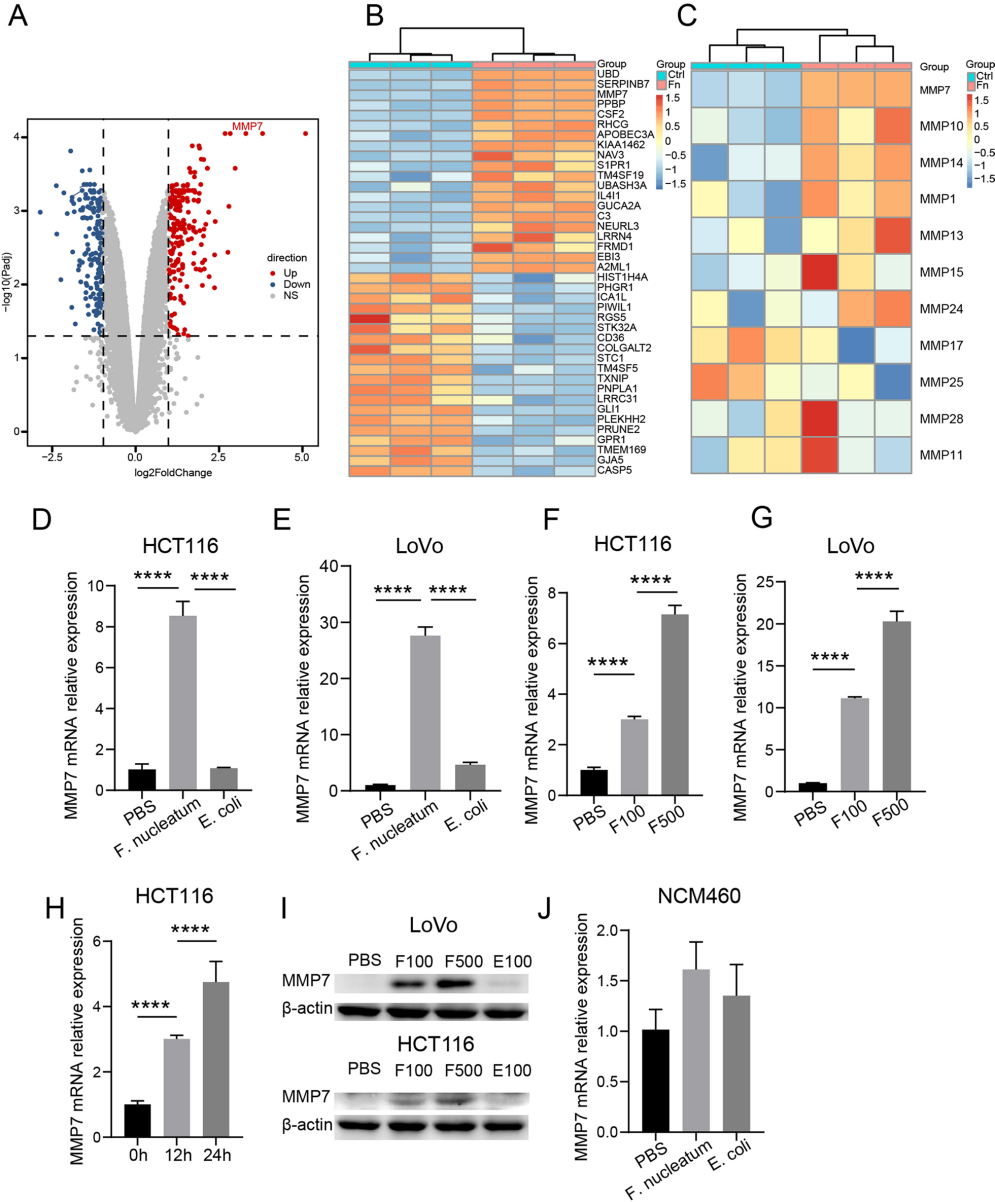
* F. nucleatum* upregulates MMP7 in CRC cells. **A**, **B** Volcano map (**A**) and heat map (**B**), representing the differentially expressed genes between *F. nucleatum*-infected and PBS-treated LoVo cell by RNA-seq (n = 3 per group, logFC ≥ 2, adjusted P value < 0.05). **C** The expression of MMP7 among all MMPs in LoVo cells incubated with *F. nucleatum* or PBS was represented by heat map (n = 3 per group). **D**, **E** The mRNA expression of MMP7 after *F. nucleatum*, *E. coli* or PBS treatment in CRC cells (n = 4 per group, One-way ANOVA test). **F**, **G** The mRNA expression of MMP7 after PBS treatment or *F. nucleatum* infection of MOI = 100 or 500 in CRC cells (n = 4 per group, One-way ANOVA test). **H** The mRNA expression of MMP7 at 0 h, 12 h, and 24 h after *F. nucleatum* infection in HCT116 cells (n = 4 per group, One-way ANOVA test). **I** The protein expression of MMP7 after PBS treatment, *F. nucleatum* infection of MOI = 100 or 500, or *E. coli* infection of MOI = 100 in CRC cells (repeated three times). **J** The mRNA expression of MMP7 after *F. nucleatum* infection in NCM460 cells (n = 4 per group). Data are shown as mean ± SD, * p < 0.05, ** p < 0.01, *** p < 0.001, **** p < 0.0001

In order to uncover the potential molecular mechanism of *F. nucleatum* promoting CRC cell migration, we performed RNA sequencing profile analysis of CRC cells with or without *F. nucleatum* infection. The result showed that MMP7 was one of the most significantly upregulated genes in *F. nucleatum*-infected LoVo cells (Fig. 3A, B). According to the report, matrix metalloproteinases play a critical role in cancer metastasis [18]. Among all the matrix metalloproteinases, MMP7 was the most upregulated matrix metalloproteinase by *F. nucleatum* infection (Fig. 3C). To confirm this find, the MMP7 expression of LoVo and HCT116 cells was analyzed by qPCR and western blot after incubation with *F. nucleatum, E. coli*, or PBS. We observed that the expression of MMP7 was significantly upregulated in *F. nucleatum*-infected CRC cells (Fig. 3D, E). Furthermore, the expression level of MMP7 rose along with the MOI or the time after infection (Fig. 3F–H). The level of protein expression was consistent with RNA expression (Fig. 3I). However, the MMP7 expression of normal colon epithelial cell NCM460 was affected by *F. nucleatum* very slightly (Fig. 3J).

### ***F. nucleatum *** promotes migration of CRC cells by upregulating MMP7

**Fig. 4:**
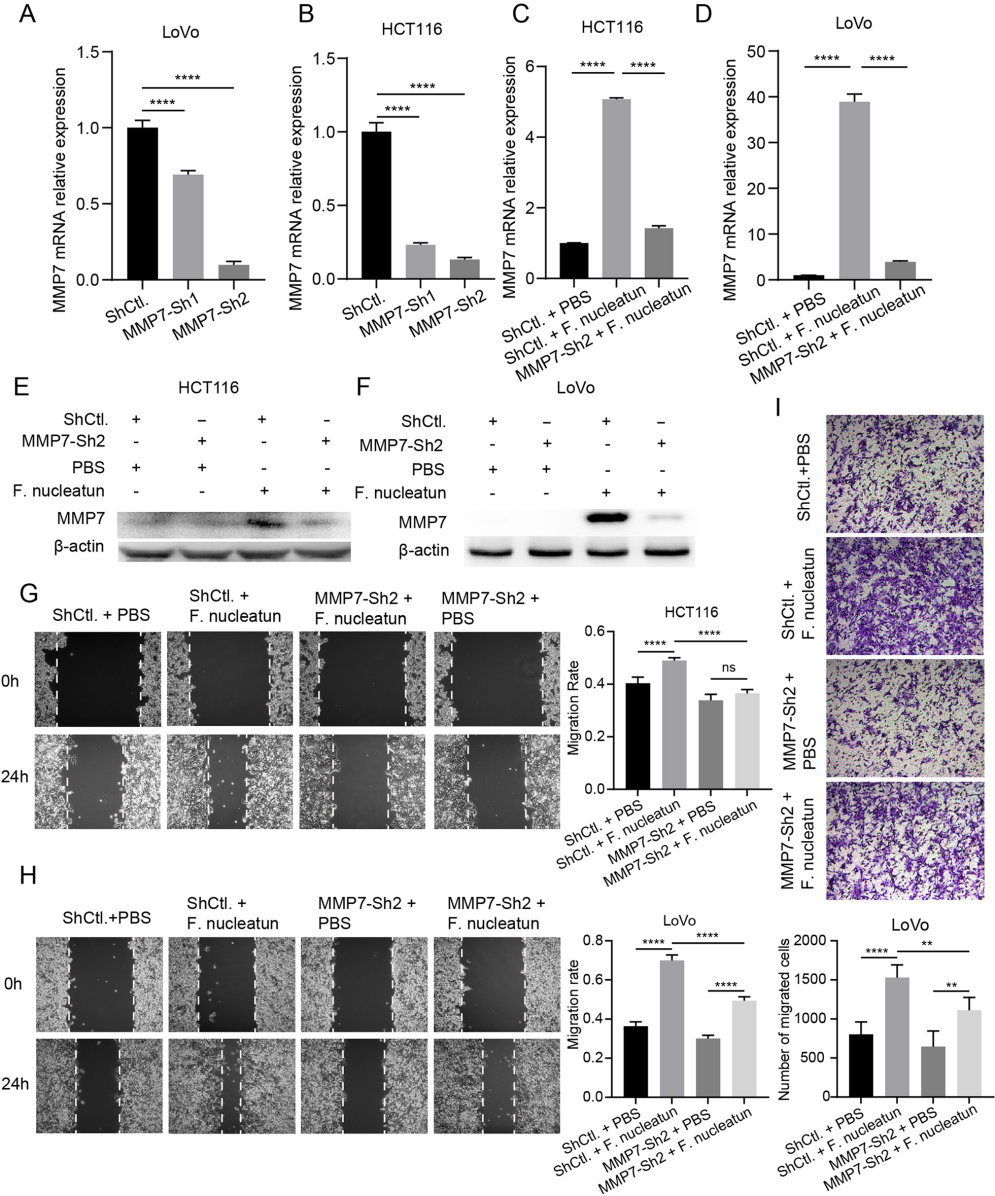
* F. nucleatum* promotes the migration of CRC cells by upregulating MMP7. **A**, **B** The mRNA expression of MMP7 in CRC cells transfected with MMP7 shRNA lentivirus or control lentivirus (n = 4). **C**–**I** CRC cells were transfected with MMP7 shRNA lentivirus or control lentivirus, and then incubated with *F. nucleatum* or PBS. The mRNA expression (**C**, **D**) and protein expression (**E**, **F**) of MMP7 were measured by qPCR (n = 4, One-way ANOVA test) and western blot (repeated three times), respectively. The motility was detected by wound-healing assay (**G**, **H**) (n = 5, One-way ANOVA test) or transwell assay (**I**) (n = 6, One-way ANOVA test). Data are shown as mean ± SD, * p < 0.05, ** p < 0.01, *** p < 0.001, p < 0.001, **** p < 0.0001

To determine whether the effect of *F. nucleatum* on the CRC cell migration was mediated by MMP7, loss-of-function assays were performed. We constructed MMP7-knockdown cell lines by using lentivirus vector. The efficiency of knockdown was verified by qPCR (Fig. 4A, B). The Lentivirus vector with knockdown efficiency of more than 50% in both two cell lines was selected for further experiments. We observed that knockdown of MMP7 attenuated *F. nucleatum*-induced upregulation of MMP7 (Fig. 4C-F), and also abolished *F. nucleatum*-enhanced migration of CRC cells (Fig. 4G–I).

### ***F. nucleatum *** upregulates MMP7 by activating MAPK (JNK)-AP1 axis

**Fig. 5:**
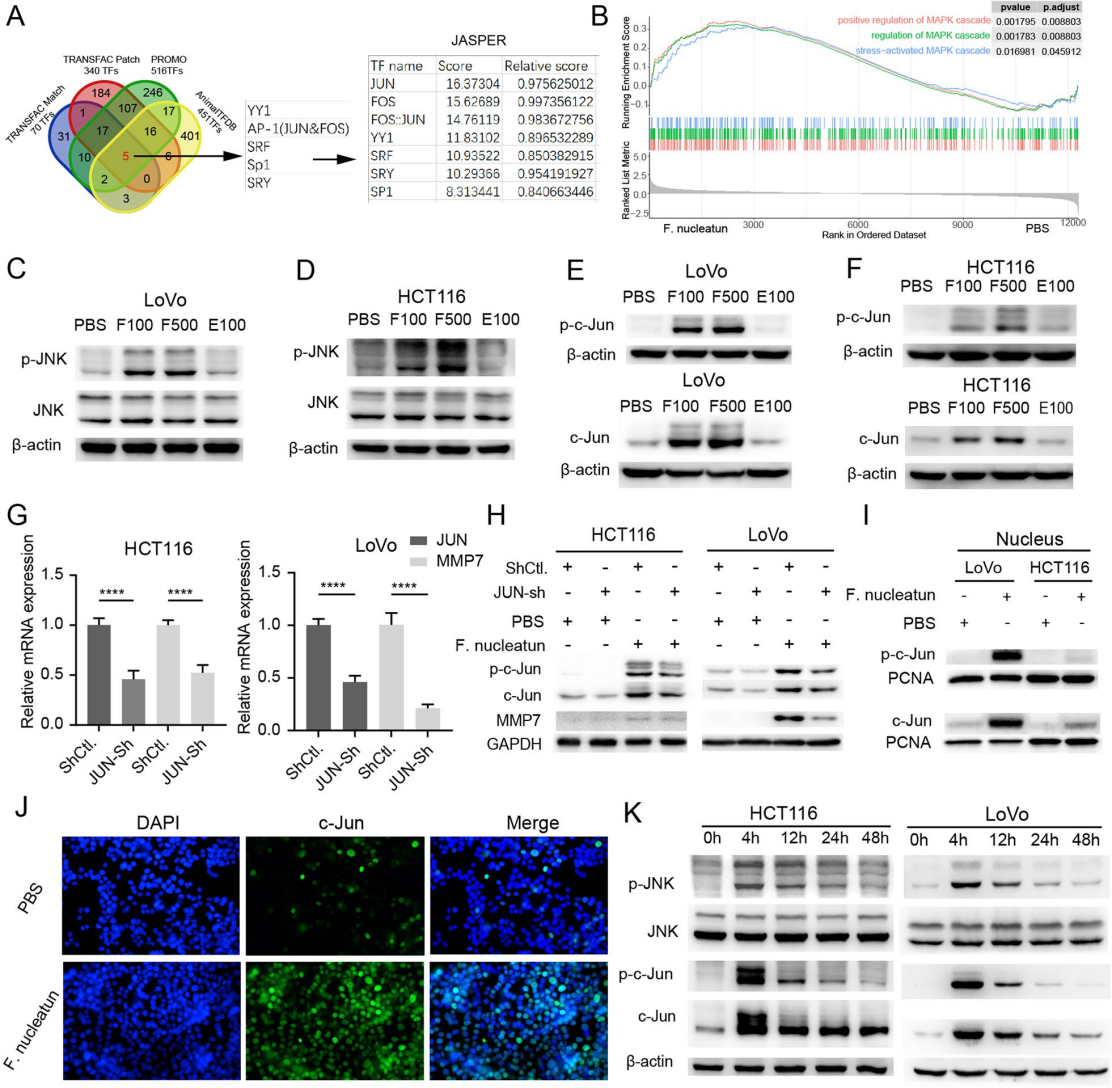
* F. nucleatum* upregulates MMP7 by activating the MAPK (JNK)-AP1 axis. **A** Venn diagram showing five overlapping transcription factors in four prediction sets (AnimalTFDB, TRANSFAC PATCH, TRANSFAC MATCH, and PROMO). The binding score of selected transcription factors was evaluated by JASPAR. **B** GSEA of RNA-seq data comparing the transcriptome of LoVo infected with or without *F. nucleatum*. **C**–**F** Protein expression of CRC cells after PBS treatment, *F. nucleatum* infection of MOI = 100 (F100) or 500 (F500), or *E. coli* infection of MOI = 100 measured by western blot (repeated three times). **G** The mRNA expression of MMP7 and JUN in CRC cells transfected with JUN shRNA lentivirus or control lentivirus (n = 4, One-way ANOVA test). **H** CRC cells transfected with JUN shRNA lentivirus or control lentivirus, were incubated with or without *F. nucleatum*. The protein expression was measured by western blot (repeated three times). **I** The protein level in the nucleus of CRC cells after *F. nucleatum* or PBS treatment measured by western blot (repeated three times). **J** LoVo cells were incubated with *F. nucleatum* or PBS for 2 h, immunofluorescence assay was conducted to show the protein c-Jun in LoVo cells (repeated three times). **K** The protein expression of CRC cells at 0 h, 4 h, 12 h, 24 h, and 48 h after *F. nucleatum* infection (repeated three times). Data are shown as mean ± SD, * p < 0.05, ** p < 0.01, *** p < 0.001, **** p < 0.0001

Using the online bioinformatics tools AnimalTFDB, TRANSFAC, PROMO and JASPAR, we predicted the potential transcription factors that may bind to the promoter region of MMP7 (Fig. 5A). Among them, AP1 subunit, JUN’s score is the highest. In addition, previous study have confirmed that AP1 is a direct transcription factor of MMP7 by Chip and luciferase reporter assays [32]. Therefore, we speculated that *F. nucleatum*-induced upregulation of MMP7 is mediated by the transcription factor AP1. By using the tool KEGG Maper, we knew that AP1 was regulated by the MAPK (JNK) pathway (Additional file 1: Fig. S1). The result of GSEA analysis indicated that *F. nucleatum* infection activated MAPK signaling (Fig. 5B). Through western blot experiment, we confirmed that JNK signaling was activated by *F. nucleatum* infection (Fig. 5C, D). *F. nucleatum* infection led to an increase in phosphorylated JNK, and its downstream proteins c-Jun and phosphorylated c-Jun also increased significantly (Fig. 5E, F). To verify the regulatory relationship between AP1 and MMP7, knockdown assays were performed. We observed that knockdown of JUN significantly suppressed the expression of MMP7 (Fig. 5G, H). In addition, we confirmed that the phosphorylated c-Jun and total c-Jun in the nucleus of CRC cell were elevated by *F. nucleatum* infection (Fig. 5I). This result was validated again by immunofluorescence experiments (Fig. 5J). We examined activation of this pathway at different time points after two hours of co-culture. We found that the signaling activation was most pronounced at 4 h after infection, and then gradually diminished over time (Fig. 5K). However, 48 h after infection, this signal was still up-regulated. To ascertain whether there is a feedback regulatory between MMP7 and its upstream proteins p-c-Jun, c-Jun, p-JNK or JNK, We detect the expression of these proteins in HCT116 and LOVO cell lines with or without MMP7 knockown and/or *F. nucleatum* infection. And We found that the expression of upstream proteins (p-JNK, JNK, p-c-Jun, c-Jun) remained unchanged after MMP7 knockdown (Additional file 2: Fig. S2). This indicates that MMP7 does not exert feedback regulation on these upstream proteins.

### MMP7 is upregulated in CRC and associated with a poor prognosis

**Fig. 6:**
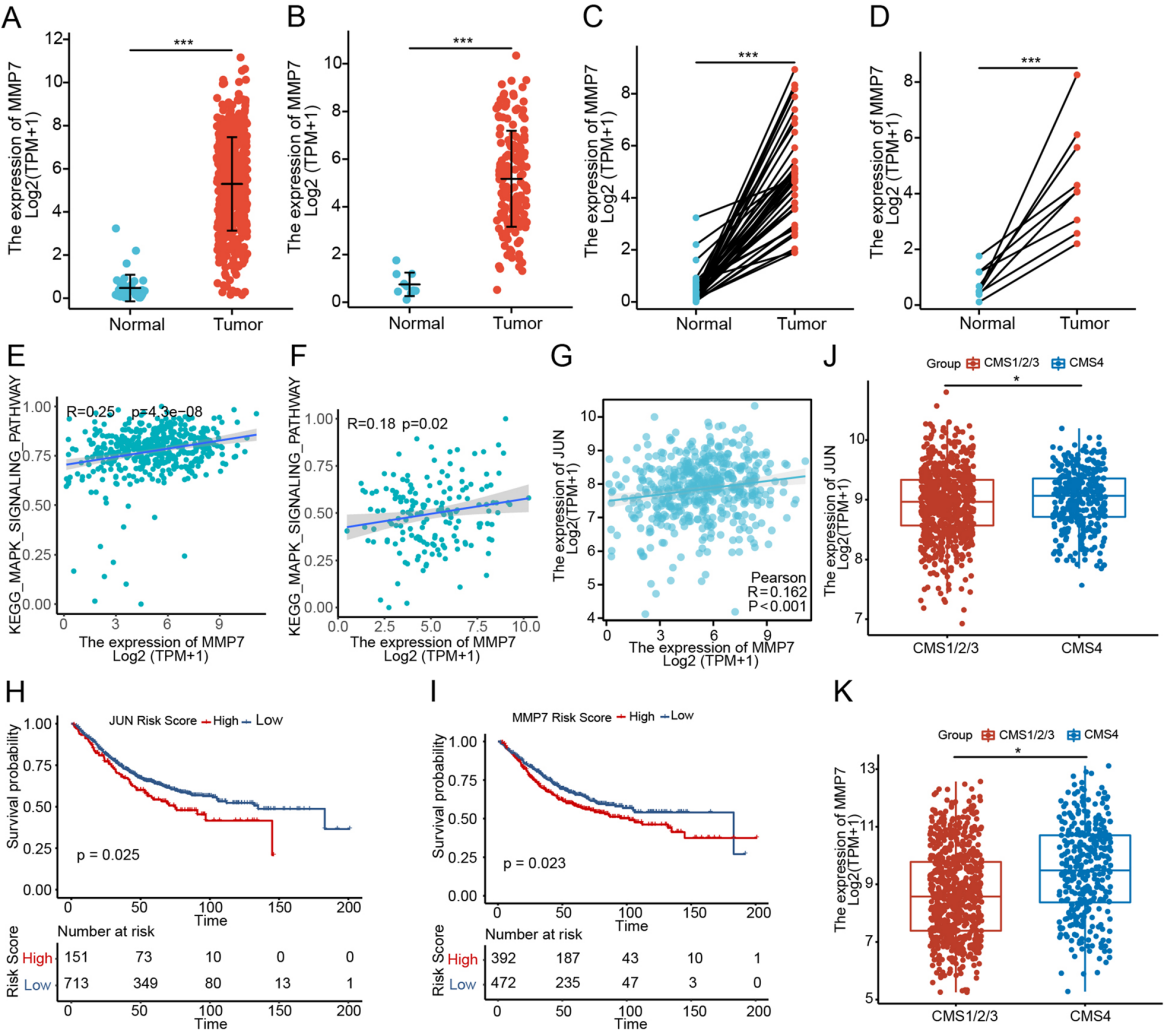
MMP7 is upregulated in CRC and associated with a poor prognosis. **A**, **B** Expression level of MMP7 in unpaired tumor tissues and normal tissues of TCGA-COAD (Normal, n = 41; Tumor, n = 480, Welch t’ test), or READ (Normal, n = 10; Tumor, n = 167, Welch t’ test). **C**, **D** Expression level of MMP7 in paired tumor tissues and adjacent normal tissues of TCGA-COAD (Normal, n = 41; Tumor, n = 41; Wilcoxon signed rank test) or READ (Normal, n = 9; Tumor, n = 9, Paired t test). **E**, **F** Pearson correlation between the MAPK signaling pathway and the expression of MMP7 in the tumor tissues from TCGA-COAD/READ (COAD, n = 471, READ, n = 165). **G** Pearson correlation between the expression of JUN and MMP7 in the tumor tissues from TCGA-COAD (n = 480). (H, I) Kaplan–Meier survival curves were analyzed and compared between patients with low and high level of JUN (**H**) and MMP7 (**I**) in CRC patients from the GEO combined dataset (n = 864, the log-rank test). **J**, **K** The JUN (**J**) and MMP7 (**K**) expression score of CMS1/2/3 (n = 713) or CMS4 (n = 340, Wilcoxon signed rank test) group in the GEO combined dataset. Data are shown as mean ± SD, * p < 0.05, ** p < 0.01, *** p < 0.001

By analyzing the gene expression of CRC patients in the TCGA-COAD/READ- datasets, we found that the mRNA expression of MMP7 in colon and rectal cancer tissues was significantly higher than normal tissues (Fig. 6A, B). The results of paired colon or rectal cancer tissues and adjacent normal tissues were similar (Fig. 6C, D). Consistent with our experimental results, MMP7 mRNA expression was positively correlated with MAPK signaling pathway in TCGA-COAD/READ (Fig. 6E, F). The mRNA expression of MMP7 was also positively correlated with JUN mRNA expression in TCGA-COAD (Fig. 6G). CRC patients with high MMP7 or JUN expression had a worse prognosis (Fig. 6H, I). The Consensus Molecular Subtypes (CMS) classification is one of the most widely used classification systems in the clinical treatment of CRC, including CMS1 (microsatellite instability immune), CMS2 (canonical), CMS3 (metabolic), and CMS4 (mesenchymal). Different subtypes of patients had different molecular characteristics, prognoses, and treatment options. CMS 4 patients were characterized by prominent transforming growth factor β (TGF-β) activation, stromal invasion, and angiogenesis with poor overall survival and recurrence-free survival [33]. According to previous research, *F. nucleatum* was prognostic for CMS4 patients [34]. Therefore, we analyzed the relationship between MMP7 and JUN and CMS classification. The results showed that the expression of MMP7 and JUN in CMS 4 patients was significantly higher than in the other three types (Fig. 6J, K). This implies the potential association between MMP7, JUN and CMS 4. It is reported that MMPs are associated with tumor epithelial-mesenchymal transformation and angiogenesis [35]. All findings support the hypothesis that *F. nucleatum*-activated JUN and MMP7 may be key molecules leading to a poor prognosis in CMS 4 patients and have the potential to correct the poor prognosis of CMS 4 patients.

### Potential small molecule drugs to target MMP7

Considering that MMP7 plays an important role in *F. nucleatum*-induced CRC cell migration, we tried to find small molecule drugs that can target MMP7 for CRC patients with high MMP7 expression. According to reports, some natural drugs have inhibitory effects on *F. nucleatum* or *F. nucleatum*-activated signal pathways in CRC [36]. We built the MMP7- components Network by Cytoscape Software according to the data of HERB database (Additional file 3: Fig. S3A). To determine the possible binding of different components to MMP7, molecular docking was performed. The heat map showed the docking score of MMP7 and different components (Additional file 3: Fig. S3B). The top four components docking with MMP7 are δ-tocotrienol, 3,4-benzopyrene, tea polyphenols, and gallic catechin. The combining details were visualized with PYMOL (Additional file 3: Fig. S3C).

## Discussion

The interactions of gut-microbiota and host have been implicated in the progression of CRC, but its underlying mechanisms remain largely unclear. Metastasis is one of the malignant features of CRC, and also a vital cause for poor prognosis of CRC patients. According to previous reports, *F. nucleatum* is overabundant in the feces and tumor tissue of CRC patients [7, 9, 37]. *F. nucleatum* abundance in the tumor is significantly higher than in neighboring normal mucosal tissue [9, 38, 39]. Tissues and feces from patients with invasive CRC (T1b-T3) contained more *F. nucleatum* than patients with early CRC (Tis and T1a) [40]. Moreover, the lymph node metastases CRC and *F. nucleatum* are positively correlated [38]. To our surprise, Bullman and colleagues proved the persistence of *F. nucleatum* in CRC distal metastases [41]. Therefore, the question whether *F. nucleatum* is the driver or the passenger of CRC progress and metastasis has puzzled many researchers and attracted them to explore this unknown field with all their passion.

By analyzing the public CRC cell line transcriptome data, we found that *F. nucleatum* infection mainly affected the biological functions of epithelial-mesenchymal transformation, cell adhesion, cadherin binding, IL-17 signaling pathway, cytoskeletal motor activity, glycolysis, and fat metabolism, all of which has been validated by pervious studies [13, 14, 42–48]. There are still some functions and mechanisms that perhaps influenced by *F. nucleatum*, but are unclear and need further exploration, such as reactive oxygen species pathway, complement cascade, hypoxia, amino acid synthesis and transmembrane transport, and ferroptosis pathways.

The mechanisms involved in the invasion and metastasis of cancer are complex and diverse, including epithelial-mesenchymal transformation, cytoskeletal remodeling, cell-cell adhesion, matrix destruction in the front of tumor invasion, and angiogenesis. Several previous studies have partially revealed the mechanisms underlying the role of Fusobacterium nucleatum in promoting CRC metastasis, such as the upregulation of metastasis-associated cytokines and chemokines, induction of epithelial-mesenchymal transition, regulation of cell adhesion-related molecules, and modulation of cytoskeletal proteins [36]. Here, our works initially found that MMP7 played a vital role in *F. nucleatum*-infection promoted CRC cell migration. MMP7, a significant member of the MMPs family, has a function in tumor growth, metastasis, and angiogenesis [18]. It can not only digest ECM macromolecule such as fibronectin, laminin, type I collagen and gelatin, but also activate other MMPs, such as pro-MMP9 and pro-MMP2 [18]. In addition, since MMP7 can degrade E-cadherin, it is speculated that MMP-7 on the cancer cell membranes cleaves E-cadherin, allowing the cell to be detached from the primary cancer cell nests [49]. Mechanistically, we revealed that *F. nucleatum* up-regulated MMP7 expression by activating MAPK (JNK) - AP1 axis. According to previous study, AP1 is a transcription factor which targets MMP7 [32]. MAPK (JNK) signal plays an important role in cell response to stress and infection [50]. F. nucleatum were likely to selectively upregulate MMP7 in CRC cells, as MMP7 expression in normal colon epithelial cell NCM460 was not significantly affected by *F. nucleatum* infection. We speculate that some upstream molecules specifically expressed in CRC cells mediate *F. nucleatum* induced activation of JNK signal. But unfortunately, we have not found such molecules. Interestingly, the up-regulation of MMP7 was *F. nucleatum*-dose-dependent, suggesting that the more amount of *F. nucleatum* in the tumor, the more significant the expression of MMP7, and the stronger the effect of *F. nucleatum* to promote cancer cell metastasis. Our study showed that MMP7 is highly expressed in CRC tissue compared to normal colorectal tissue and is associated with poor prognosis of patients. This finding was consistent with the previous study [51]. Small molecular drugs play an important role in the field of cancer targeted therapy. Some compounds, such as berberine, L-fucose, vanillin derivatives, TAK-242, have been used in targeted therapy for *F. nucleatum* and its activated signal pathway in pilot study [36]. We tried to screen small molecular agents targeting MMP7 through using HERB dataset and molecular docking. The result showed that δ-tocotrienol, 3,4-benzopyrene, tea polyphenols, and gallic catechin had a high binding score with MMP7 protein, and are potential targeted therapeutic drugs for *F. nucleatum* infected CRC patients.

There are still some deficiencies in our study. Experiments in vivo need to be carried out to further verify our theory. We lack large size clinical cohort to validate the correlation between *F. nucleatum* and the expression level of MMP7 and c-Jun in CRC. We also lack further experiments to confirm the inhibitory effect of small molecular drugs on the MMP7 protein and migratory capacity of CRC cells.

## Conclusion

Taken together, our results suggested that *F. nucleatum* infection altered gene expression profile and biological function in CRC. *F. nucleatum* was overabundant in CRC and upregulated MMP7 to accelerate CRC cell migration via activating MAPK(JNK)-AP1 axis. MMP7 and *F. nucleatum* may serve as potential therapeutic targets for patients with CRC.

### Supplementary Information

**Additional file 1: Figure S1.** KEGG Maper showing the regulatory relationships among JNK, AP1, and MMP7.

**Additional file 2: Figure S2. A**, **B** CRC cells were transfected with MMP7 shRNA lentivirus or control lentivirus, and then incubated with F. nucleatum or PBS. The protein expression were measured by western blot (repeated three times).

**Additional file 3: Figure S3. A** Combination network of MMP7 and different components. **B** Heat map showing the molecular docking scores of different components and MMP7. **C** Molecular docking model of different components with MMP7.

## Data Availability

The data used in this study are available from the corresponding author upon request.
