# Supplementary figures and images for: Fusobacterium nucleatum upregulates MMP7 to promote metastasis-related characteristics of colorectal cancer cell via activating MAPK(JNK)-AP1 axis
Source: J Transl Med. 2023 Oct 9;21:704. doi: 10.1186/s12967-023-04527-3 (PMC10561506; doi:10.1186/s12967-023-04527-3)

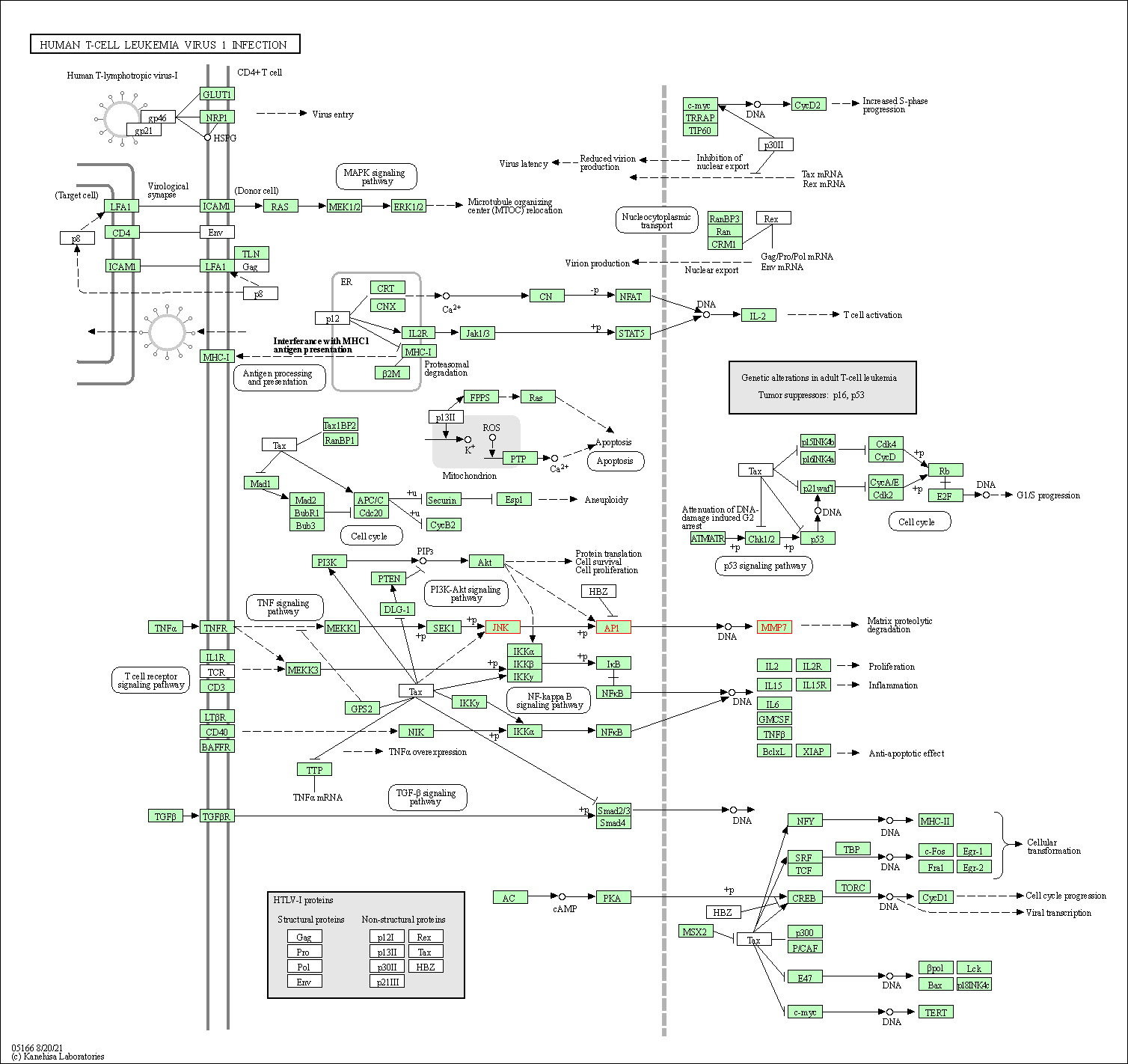


**Figure S1.** KEGG Maper showing the regulatory relationships among JNK, AP1, and MMP7.

Supplement: Supplementary file 1 — Additional file 1: Figure S1. KEGG Maper showing the regulatory relationships among JNK, AP1, and MMP7. [file 12967_2023_4527_MOESM1_ESM.docx]
