# Supplementary material for: Fusobacterium nucleatum upregulates MMP7 to promote metastasis-related characteristics of colorectal cancer cell via activating MAPK(JNK)-AP1 axis
Source: J Transl Med. 2023 Oct 9;21:704. doi: 10.1186/s12967-023-04527-3 (PMC10561506; doi:10.1186/s12967-023-04527-3)
Supplement: Supplementary file 2 — Additional file 2: Figure S2. A, B CRC cells were transfected with MMP7 shRNA lentivirus or control lentivirus, and then incubated with F. nucleatum or PBS. The protein expression were measured by western blot (repeated three times). [file 12967_2023_4527_MOESM2_ESM.docx]

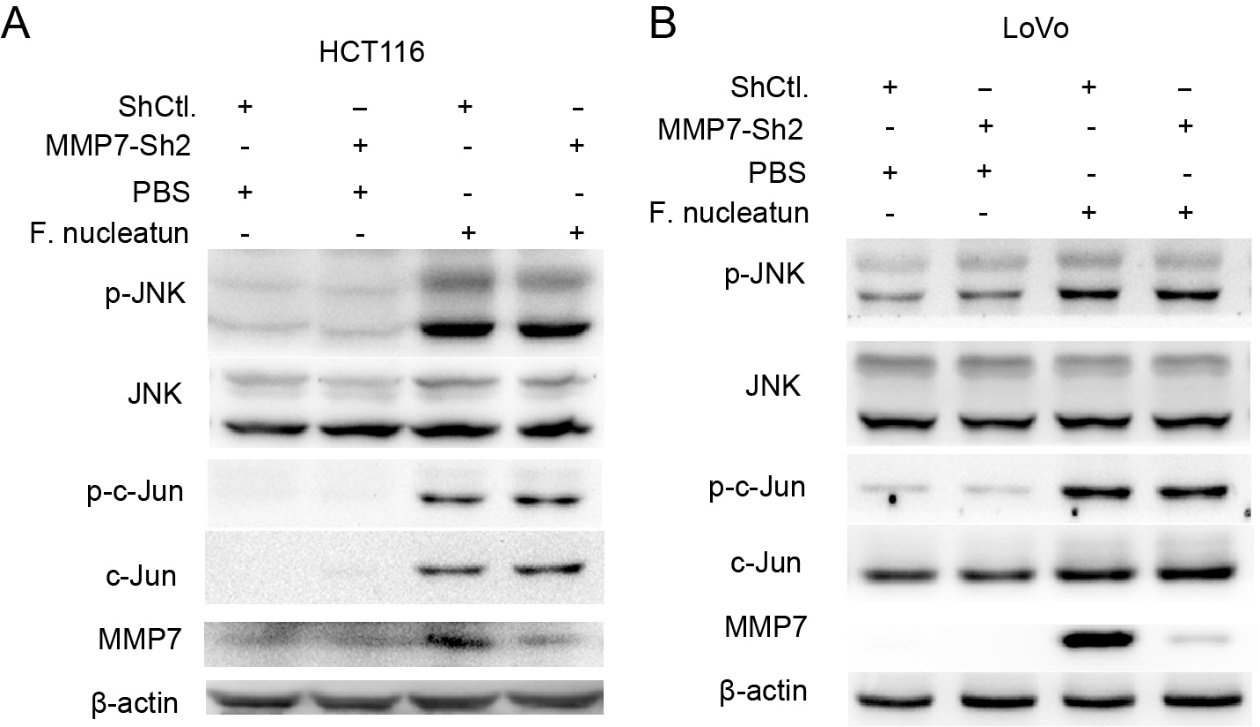


**Figure S2.** (A, B) CRC **c**ells were transfected with MMP7 shRNA lentivirus or control lentivirus, and then incubated with *F. nucleatum* or PBS. The protein expression were measured by western blot (repeated three times).
