# Supplementary material for: Fusobacterium nucleatum upregulates MMP7 to promote metastasis-related characteristics of colorectal cancer cell via activating MAPK(JNK)-AP1 axis
Source: J Transl Med. 2023 Oct 9;21:704. doi: 10.1186/s12967-023-04527-3 (PMC10561506; doi:10.1186/s12967-023-04527-3)
Supplement: Supplementary file 3 — Additional file 3: Figure S3. A Combination network of MMP7 and different components. B Heat map showing the molecular docking scores of different components and MMP7. C Molecular docking model of different components with MMP7. [file 12967_2023_4527_MOESM3_ESM.docx]

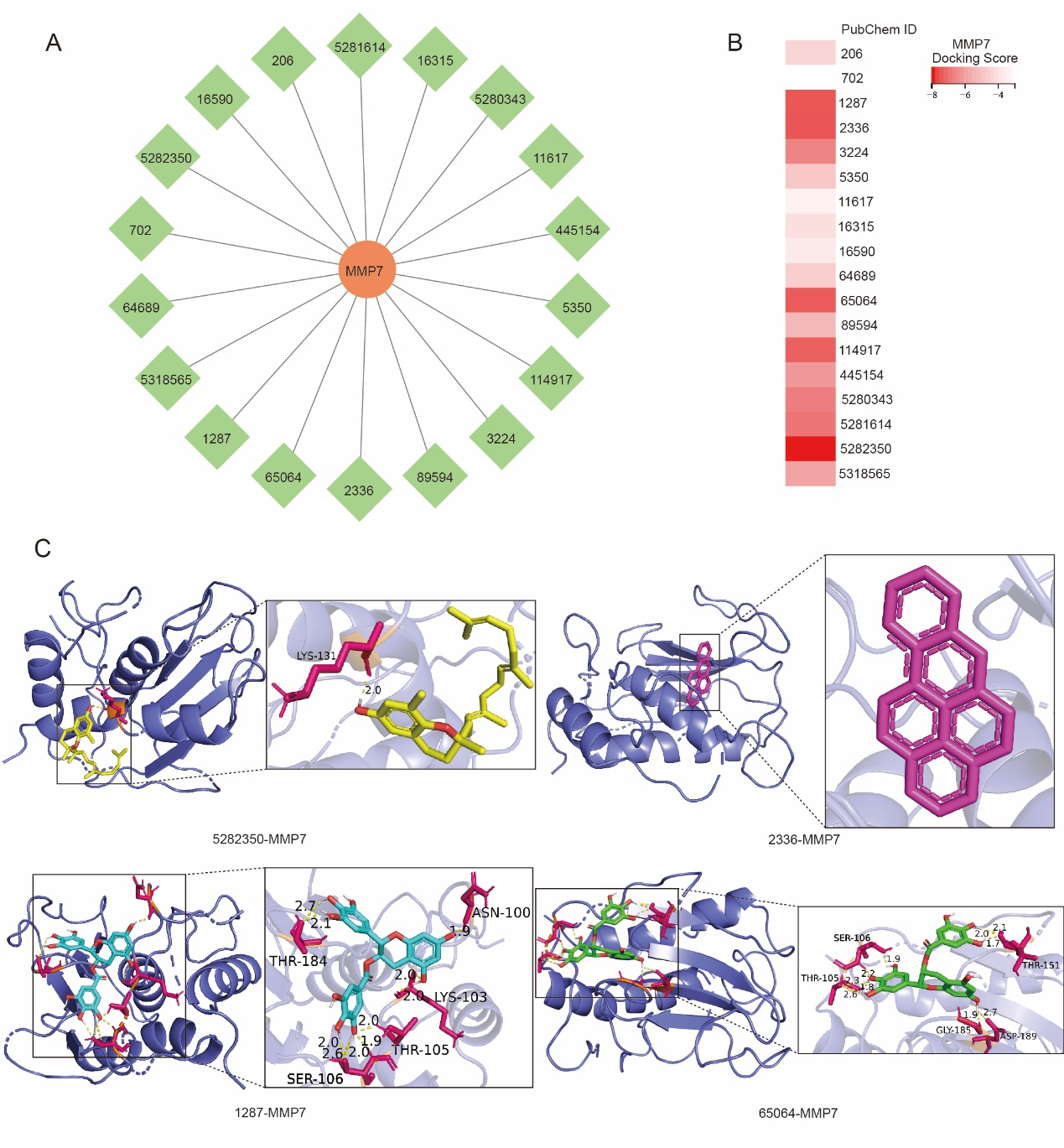


**Figure S3.** (A) Combination network of MMP7 and different components. (B) Heat map showing the molecular docking scores of different components and MMP7. (C) Molecular docking model of different components with MMP7.
